# Analyzing Illumina Gene Expression Microarray Data from Different Tissues: Methodological Aspects of Data Analysis in the MetaXpress Consortium

**DOI:** 10.1371/journal.pone.0050938

**Published:** 2012-12-07

**Authors:** Claudia Schurmann, Katharina Heim, Arne Schillert, Stefan Blankenberg, Maren Carstensen, Marcus Dörr, Karlhans Endlich, Stephan B. Felix, Christian Gieger, Harald Grallert, Christian Herder, Wolfgang Hoffmann, Georg Homuth, Thomas Illig, Jochen Kruppa, Thomas Meitinger, Christian Müller, Matthias Nauck, Annette Peters, Rainer Rettig, Michael Roden, Konstantin Strauch, Uwe Völker, Henry Völzke, Simone Wahl, Henri Wallaschofski, Philipp S. Wild, Tanja Zeller, Alexander Teumer, Holger Prokisch, Andreas Ziegler

**Affiliations:** 1 Interfaculty Institute for Genetics and Functional Genomics, Ernst-Moritz-Arndt-University Greifswald, Greifswald, Germany; 2 Institute of Human Genetics, Helmholtz Zentrum München, German Research Center for Environmental Health, Neuherberg, Germany; 3 Institut für Medizinische Biometrie und Statistik, Universität zu Lübeck, Universitätsklinikum Schleswig-Holstein, Campus Lübeck, Lübeck, Germany; 4 Klinik für Allgemeine und Interventionelle Kardiologie, Universitäres Herzzentrum Hamburg, Hamburg, Germany; 5 DZHK (German Centre for Cardiovascular Research), partner site Hamburg/Kiel/Lübeck, Hamburg, Germany; 6 Institute for Clinical Diabetology, German Diabetes Center, Leibniz Center for Diabetes Research at Heinrich Heine University Düsseldorf, Düsseldorf, Germany; 7 Department of Internal Medicine B, University Medicine Greifswald, Greifswald, Germany; 8 DZHK (German Centre for Cardiovascular Research), partner site Greifswald, Greifswald, Germany; 9 Institute of Anatomy and Cell Biology, University Medicine Greifswald, Greifswald, Germany; 10 Institute of Genetic Epidemiology, Helmholtz Zentrum München, German Research Center for Environmental Health, Neuherberg, Germany; 11 Research Unit of Molecular Epidemiology, Helmholtz Zentrum München, German Research Center for Environmental Health, Neuherberg, Germany; 12 Institute for Community Medicine, University Medicine Greifswald, Greifswald, Germany; 13 Medical School Hannover, Hannover Unified Biobank, Hannover, Germany; 14 DZHK (German Centre for Cardiovascular Research), partner site Munich, Munich, Germany; 15 Institut für Humangenetik, Technische Universität München, München, Germany; 16 Munich Heart Alliance, Munich, Germany; 17 Institute of Clinical Chemistry and Laboratory Medicine, University Medicine Greifswald, Greifswald, Germany; 18 Institute of Epidemiology II, Helmholtz Zentrum München, German Research Center for Environmental Health, Neuherberg, Germany; 19 Institute of Physiology, University Medicine Greifswald, Karlsburg, Germany; 20 Department of Metabolic Diseases, University Hospital Düsseldorf, Heinrich-Heine University, Düsseldorf, Germany; 21 Institute of Medical Informatics, Biometry and Epidemiology, Chair of Genetic Epidemiology, Ludwig-Maximilians-Universität, Munich, Germany; 22 Center for Thrombosis and Hemostasis, University Medical Center of the Johannes Gutenberg-University Mainz, Mainz, Germany; 23 Department of Medicine 2, University Medical Center of the Johannes Gutenberg-University Mainz, Mainz, Germany; 24 DZHK (German Centre for Cardiovascular Research), partner site Rhine-Main, Mainz, Germany; 25 DZHK (German Centre for Cardiovascular Research), partner site Hamburg/Kiel/Lübeck, Lübeck, Germany; Ernst-Moritz-Arndt-University Greifswald, Germany

## Abstract

Microarray profiling of gene expression is widely applied in molecular biology and functional genomics. Experimental and technical variations make meta-analysis of different studies challenging. In a total of 3358 samples, all from German population-based cohorts, we investigated the effect of data preprocessing and the variability due to sample processing in whole blood cell and blood monocyte gene expression data, measured on the Illumina HumanHT-12 v3 BeadChip array.

Gene expression signal intensities were similar after applying the log_2_ or the variance-stabilizing transformation. In all cohorts, the first principal component (PC) explained more than 95% of the total variation. Technical factors substantially influenced signal intensity values, especially the *Illumina chip* assignment (33–48% of the variance), the *RNA amplification batch* (12–24%), the *RNA isolation batch* (16%), and the sample *storage time*, in particular the *time between blood donation and RNA isolation* for the whole blood cell samples (2–3%), and the *time between RNA isolation and amplification* for the monocyte samples (2%). White blood cell composition parameters were the strongest biological factors influencing the expression signal intensities in the whole blood cell samples (3%), followed by sex (1–2%) in both sample types. Known single nucleotide polymorphisms (SNPs) were located in 38% of the analyzed probe sequences and 4% of them included common SNPs (minor allele frequency >5%). Out of the tested SNPs, 1.4% significantly modified the probe-specific expression signals (Bonferroni corrected p-value<0.05), but in almost half of these events the signal intensities were even increased despite the occurrence of the mismatch. Thus, the vast majority of SNPs within probes had no significant effect on hybridization efficiency.

In summary, adjustment for a few selected technical factors greatly improved reliability of gene expression analyses. Such adjustments are particularly required for meta-analyses.

## Introduction

Global gene expression studies are widely conducted in molecular biology and functional genomics [1]. They have successfully provided new insights into the etiology of common diseases [2]. Especially for cancer, gene expression profiling is already used in medical applications, such as the identification of breast cancer disease subgroups using the intrinsic subtype classifier [3] or the prognosis of breast-cancer survival using MammaPrint [4], [5]. Furthermore, gene expression analysis is applied in clinical trials to examine drug response [6] (for an overview, see [7]) and some gene expression profiles have already been cleared by the US Food and Drug Administration (FDA) as in vitro diagnostic multiple index assay, now generally termed companion diagnostic, to be used as predictive biomarkers for guiding treatment decision [8], [9].

Although gene expression studies have been successfully applied to a wide range of clinical issues, they are often criticized for low robustness and lack of reproducibility [10], [11]. Concerns also include improper statistical analysis or validation, insufficient control of false positives, and inadequate reporting of methods [1]. As a consequence, virtually all major journals have adopted standards for the conduct and reporting of microarray experiments [12].

When multiple independent gene expression studies are available, their combined analysis or even a meta-analysis can increase the reliability and generalizability of the results [1]. Another important advantage of combined analyses or meta-analyses is the increase in statistical power. Furthermore, the combination of several studies may help to identify and to better understand heterogeneity between studies. To this end, meta-analyses have been performed on studies covering a wide range of diseases, ranging from various cancers [13], [14], [15], [16] to very rare diseases including intracranial aneurysms [17] and systemic lupus erythematosus [18]. Recently, several methodological developments have been made to facilitate meta-analyses of gene expression studies [19], [20] and several web resources are now available [21], [22], [23], [24]. Despite these developments meta-analyses of global gene expression studies remain challenging. The issues encountered include problems common to traditional meta-analyses [25], such as differences in study design, as well as concerns that are specific for analyzing gene expression data [1], [26]. One of the latter aspects is related to the technology used. Specifically, different types of microarrays vary fundamentally in important aspects, such as length of probes, scale of measurements or coefficients of variation [1], [26]. As a result, cross-platform comparisons are difficult to perform.

Even if several independent studies use the same microarray technology, there may be study-specific laboratory effects, originating from differences in experimental procedures, laboratory protocols, sample preparation [1], [26], or type of tissue [27]. In addition, different preprocessing of the data as well as batch effects, e.g. due to grouped sample processing, may lead to differences in measured gene expression levels in large sample size studies.

Recently, we have established the MetaXpress (Meta-Analysis of Gene Expression) Consortium within the German Center for Cardiovascular Disease (DZHK) to facilitate the meta-analysis of gene expression studies. Members of the consortium are three population-based cohorts, the Study of Health in Pomerania (SHIP-TREND) [28], the Cooperative Health Research in the Region of Augsburg (KORA F4) [29], and the Gutenberg Health Study (GHS) [30]. In all three studies, gene expression levels in terms of mRNA abundances were measured using BeadChip arrays (Illumina, HumanHT-12 v3). The gene expression data were generated from whole blood cells (SHIP-TREND, n = 991 and KORA F4, n = 993) or from blood monocytes (GHS, n = 1374).

In the present study, we investigated the influence of data preprocessing and technical factors related to sample processing on measured gene expression levels in whole blood cells or blood monocytes. First, we compared the log_2_ transformation (L2T) of intensity values [31] with the recently proposed variance-stabilizing transformation (VST) [32]. Next, we searched for main factors correlating with the overall expression levels. Since within study variation is often corrected for by adjusting for principal components (PC), we analyzed the correlation between the PCs and technical as well as biological factors. Our data demonstrate that the variation of gene expression signal intensities can be reduced by appropriate technical covariate adjustment. Previously, doubts have been raised about the suitability of using probes containing single nucleotide polymorphisms (SNPs) in gene expression studies [33]. We therefore investigated to what extent signal intensities were affected by mismatch alleles of SNPs within probes. Furthermore, we discuss how gene expression levels will be compared between the different studies in a Supplement (Text S1). Finally, we provide a probe annotation file based on transcript mapping.

## Results

### Study description

In this project, we analyzed gene expression levels in terms of specific mRNA abundances measured in whole blood cell (SHIP-TREND and KORA F4) or blood monocyte samples (GHS). The descriptive statistics of the participants and parameters analyzed in the study are provided in Table 1 and in Table S1. We investigated the effects of body mass index (BMI) as an example phenotype which is known to be strongly associated with gene expression profiles in whole blood cells [34] and monocytes [30]. Furthermore, we analyzed a pseudo-phenotype generated by selecting random values from a standard normal distribution. This so-called random phenotype is free of any correlation with or confounding effects of technical parameters related to the arrays or sample phenotypes.

**Table 1:** Cohort characteristics.

Variable (mean/SD)	SHIP-TREND	KORA F4	GHS
Sample size	991	993	1374
*Storage time* * [days]	204.0±153.8	855.5±179.4	314.4±91.6
*RNA integrity number*	8.56±0.50	8.68±0.61	9.36±0.43
Females (%)	555 (56.0)	493 (49.6)	622 (48.4)
Age [years]	50.1±13.7	70.4±5.4	54.7±11.0
Body height [cm]	169.8±9.0	165.3±8.8	171.0±9.3
Body weight [kg]	79.0±15.1	78.9±13.7	79.1±15.5
Body mass index [kg/m^2^]	27.3±4.6	28.9±4.5	27.0±4.6
Hip circumference [cm]	101.3±9.6	107.8±9.3	100.5±9.6
Waist circumference [cm]	88.0±12.9	98.6±12.1	93.5±13.4
Waist-to-hip ratio	0.87±0.09	0.91±0.08	0.93±0.09
White blood cell count [Gpt/l]	5.72±1.48	6.00±1.80	7.04±3.81
Red blood cell count [Tpt/l]	4.63±0.39	4.50±0.40	4.69±0.41
Hematocrit	0.42±0.03	0.41±0.03	0.42±0.03
Hemoglobin [mmol/l]	8.62±0.74	8.69±0.75	9.10±0.74
Platelets [Gpt/l]	225.7±50.3	244.7±65.1	271.5±67.9
Serum C-reactive protein [mg/l]	-	3.05±6.27	3.78±4.92
High density lipoprotein [mmol/l]	1.48±0.37	1.43±0.36	1.47±0.40
Serum triglycerides [mmol/l]	1.42±0.85	1.50±0.84	1.46±0.91
Active smokers [%]	214 (22.0)	66 (6.7)	239 (18.6)
Systolic blood pressure [mmHg]	124.4±16.9	128.7±20.0	132.2±17.8
Diastolic blood pressure [mmHg]	76.6±9.8	74.0±10.1	83.5±9.68

* *Storage time*: *Time between blood donation and RNA isolation* (SHIP-TREND and KORA F4) *or time between RNA isolation and RNA amplification* (GHS).

A dash indicates that the variable was not available in the cohort.

### Comparison of log2 and variance-stabilizing transformation

**Figure 1:**
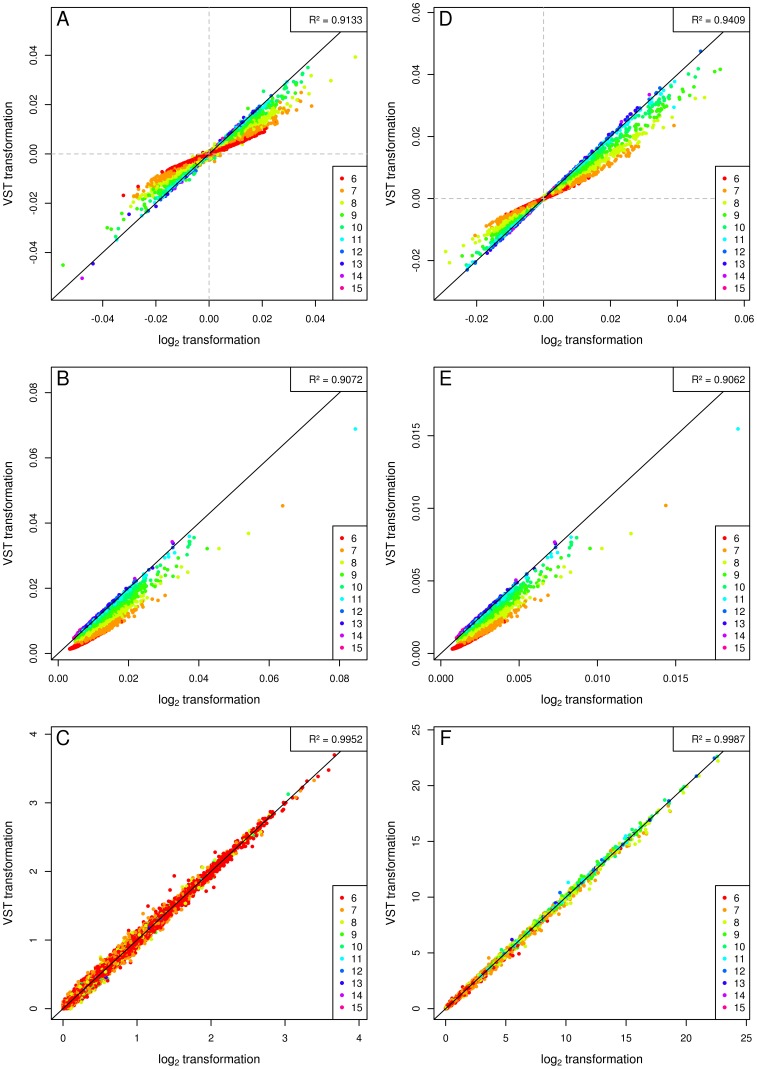
Log_2_ transformation (L2T) versus variance-stabilizing transformation (VST). The panels show the association results for the random phenotype (A–C) and for body mass index (BMI) (D–F) on each mRNA probe adjusted for sex, age, *RNA amplification batch*, *RNA integrity number* (*RIN*) and the sample *storage time* based on L2T expression values (x-axis) and on VST values (y-axis) in the SHIP-TREND cohort. The upper panels (A, D) show the betas, the middle panels (B, E) show the standard errors (SEs) and the lower panels (C, F) show the negative log_10_ association p-values. The corresponding squared Pearson product-moment correlation coefficient between the plotted values is given in the upper right corner of each plot. Each spot represents a probe and is colored according to its mean L2T expression value from all samples. The color code is given in the legend located in the lower right corner of each plot. Although betas and SEs differ between both transformations, the association p-values are highly correlated.

To assess possible differences between different data preprocessing steps, we compared L2T signal intensity data with those obtained after VST as implemented in the lumi Bioconductor package [31]. L2T data were almost equal to the VST data for signal intensities greater than 2^9^, but recognizably smaller for low intensity values, which corresponds to results published before (Figure S1) [32]. Both the absolute effect sizes and standard errors (SEs) were smaller with VST data than with L2T data for low signal intensities resulting in similar association p-values across the whole intensity spectrum for both, the body mass index (BMI) and the random phenotype (Figure 1, Figure S2).

### Main factors influencing overall gene expression data

To unravel components having a large influence on the overall expression signal intensity values, we conducted a principal component analysis (PCA). In the data sets from all three studies, the first PC accounted for approximately 96% and the first 10 PCs accounted for more than 97% of the total variation. These results were essentially unaffected by the transformation method and by the exclusion of low intensity probes that were not significantly expressed above the background level (according to the Illumina GenomeStudio software, detection p-value≥0.01 in at least 50% of the samples) (Figure S3). To identify specific parameters correlating with the measured expression profiles, we used the PCA-based *Eigen-R^2^* algorithm [35], which estimates the proportion of the expression profiles' variance explained by predefined factors. We selected 66 technical and biological factors that were available in SHIP-TREND (Table S2). Among these factors, the technical parameters *Illumina chip* (33.7%), *RNA amplification batch* (20.2%), and *RNA isolation batch* (16.5%) had the strongest effects on the gene expression profiles, whereas *storage time* of the samples (*time between blood donation and RNA isolation*, 2.9%) and the *RNA integrity number* (*RIN*) (1.4%) had only a minor effect. Biological factors of notable effect were white blood cell composition parameters of the whole blood samples, such as percentage of lymphocytes and neutrophils (2.8% and 2.7%, respectively), followed by sex (0.9%), serum magnesium concentrations (0.9%), somatometric parameters (<0.8%) including BMI (0.7%), serum triglyceride concentrations (0.7%) and age (0.6%). The results were similar across studies for variables available in multiple data sets (Table 2, Table S3) with some minor exceptions. Differences between the whole blood cell and monocytes samples were observed for the effects of hemoglobin and the percentages of lymphocytes and neutrophils as well as for other blood cell-related parameters as expected due to the different blood cell types analyzed. There was a high *Eigen-R^2^* value for the *month of blood donation* in SHIP-TREND. This can be explained by the grouping of blood samples into array processing batches consecutively by their date of blood collection in this sample. The *month of blood donation* was therefore highly correlated with both the *RNA amplification batch* and the *RNA isolation batch*.

**Table 2:** *Eigen-R^2^* results for SHIP-TREND, KORA F4 and GHS.

	*Eigen-R^2^*		
Parameter	SHIP-TREND	KORA F4	GHS
*Illumina Chip* (12 arrays per chip)	33.75%	48.18%	26.55%
*RNA amplification batch* (96 well plate)	20.18%	24.30%	12.44%
*Storage time* * [days]	2.86%	1.60%	1.70%
*Month of blood donation*	18.72%	3.31%	8.11%
*Time of blood donation* [h]	0.20%	0.41%	0.61%
*RNA integrity number*	1.36%	0.77%	0.29%
Sex	0.95%	0.87%	1.51%
Age [years]	0.58%	0.45%	0.30%
Body height [cm]	0.54%	0.48%	0.82%
Body weight [km]	0.59%	0.60%	0.51%
Body mass index [kg/m^2^]	0.68%	0.54%	0.35%
Hip circumference [cm]	0.60%	0.41%	0.27%
Waist circumference [cm]	0.77%	0.67%	0.52%
Waist to hip ratio	0.65%	0.70%	0.82%
White blood cell count [Gpt/l]	0.89%	0.74%	0.23%
Red blood cell count [Tpt/l]	0.38%	0.35%	0.65%
Hematocrit	0.47%	0.46%	0.83%
Hemoglobin [mmol/l]	0.50%	0.42%	1.03%
Platelets [Gpt/l]	0.32%	0.27%	0.63%
High density lipoprotein [mmol/l]	0.49%	0.48%	0.48%
Serum triglycerides [mmol/l]	0.68%	0.87%	0.23%
Active smokers [%]	0.36%	0.23%	0.26%
Systolic blood pressure [mmHg]	0.41%	0.15%	0.26%
Diastolic blood pressure [mmHg]	0.37%	0.14%	0.19%
Serum C-reactive protein [mg/l]	-	0.30%	0.26%

* *Storage time*: *Time between blood donation and RNA isolation* (SHIP-TREND and KORA F4) or *time between RNA isolation and RNA amplification* (GHS).

The first six lines of the Table represent technical parameters. A dash indicates that the parameter was not available in the cohort.

### Correlation of available parameters with PCs

In an attempt to identify the technical and biological underpinnings of the PCs, we correlated the first 50 PCs with 66 selected factors that were available in SHIP-TREND. The technical parameters that explained most of the variance of the measured gene expression levels, i.e., the *Illumina chip* assignment, the *RNA amplification batch*, and the *RNA isolation batch*, were highly correlated with almost all PCs. The strongest association of these three parameters with a PC was observed for the second one (p<10^−30^). Altogether, 26 factors had their lowest association p-value (Bonferroni corrected p<0.05/66 = 7.6×10^−4^) with one of the first five PCs: Sample *storage time*, *RIN*, serum concentration of magnesium, calcium and potassium, parameters related to the type of blood cell composition, such as white blood cell count (WBC), and percentage of lymphocytes and neutrophils, anthropometric parameters such as BMI, waist and hip circumference and body weight, metabolic parameters such as vitamin B12, triglycerides and high-density lipoproteins as well as serum concentration of intracellular enzymes associated with blood group antigens (alanine aminotransferases, lactate dehydrogenase and lipase) as well as regulatory factors such as partial thromboplastin time (Table S2). These factors were tested in KORA F4 and GHS if available. The technical factors showed similar association patterns with the PCs except for *RIN*, which was measured before storage of the RNA samples in GHS and after the storage of blood samples and subsequent isolation of RNA in both, SHIP-TREND and KORA F4 (Figure S4). The patterns of the biological factors varied between the studies and even more so between samples obtained from whole blood cells and those obtained from monocytes.

### Variance reduction by covariate adjustment

**Figure 2:**
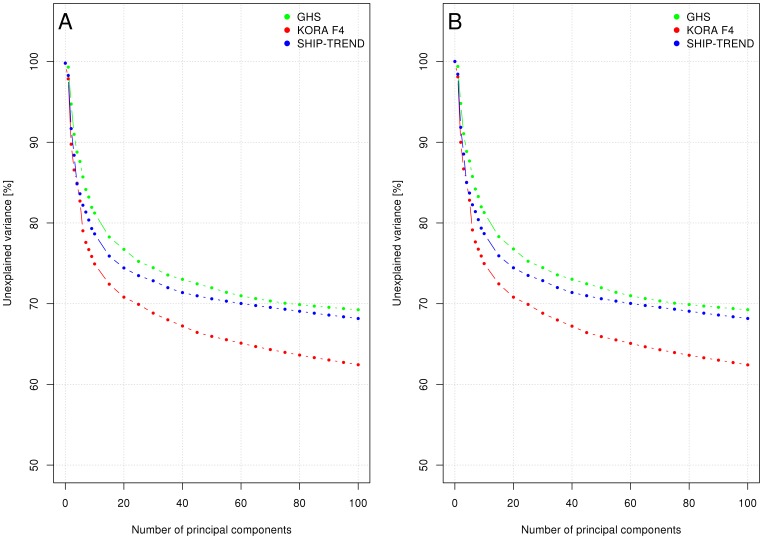
Unexplained variance after adjustment for principle components (PCs). The panels show the percentage of adjusted unexplained variance (y-axis) of the regression on the log_2_ transformed (L2T) gene expression levels and body mass index (BMI) (A) or the random phenotype (B) over the first 100 PCs (x-axis). With both phenotypes the unexplained variance decreases continuously with the addition of further PCs to the regression model. Results are given separately for the SHIP-TREND, KORA F4 and GHS cohorts.

Adding the first 50 PCs as covariates to the regression models of the gene expression levels for both the random phenotype and BMI reduced the unexplained variance by approximately 30% (Figure 2). The information obtained from PCA-based analyses was used to increase the statistical power of association analyses by reducing the residual variance when regressing the probes' expression values on a phenotype of interest. In detail, we compared the mean overall unadjusted effects, SEs and association p-values of the BMI and the random phenotype with the respective values after adding the technical and biological parameters or PCs as additional covariates into the linear regression model. For the random phenotype, the lowest mean SE was achieved by adding the first 50 PCs as covariates, thereby reducing the SEs by 21%, 27% and 25% compared with the unadjusted models in SHIP-TREND, KORA F4 and GHS, respectively (Table 3). Since some PCs could be correlated with the phenotype of interest, effect estimates of true associations might converge to zero using this approach. Therefore, in most scenarios adjustments for well-defined parameters will be preferable to a PC adjustment. By adjusting for *RNA amplification batch*, *RIN* and the sample *storage time* (*time between blood donation and RNA isolation* in SHIP-TREND and KORA F4 or *time between RNA isolation and amplification* in GHS), the SEs in both the BMI and the random phenotype association analyses were reduced by more than 8%. Additionally adding sex, age, and parameters related to white blood cell composition (percentage of lymphocytes, neutrophils, monocytes, eosinophils, and basophils) or even adding all biological parameters with an *Eigen-R^2^* value>0.3% in SHIP-TREND led only to marginal reductions in mean SEs or even slightly increased SEs in random phenotype associations (Table 3, Figure S5). As expected, when adjusting the regression of gene expression intensities on BMI for correlated parameters, the effect estimates of most associations were reduced and close to zero, the SEs changed substantially, and the p-values increased (Figure S5). An increase in the mean SE was also observed after adjustment for sex and age in the BMI regression model compared to the unadjusted model.

**Table 3:** Mean standard errors (SEs) for SHIP-TREND, KORA F4 and GHS after different covariate adjustments for the random phenotype and body mass index (BMI).

		Mean SE		
Phenotype	additional covariates (besides phenotype)	SHIP-TREND	KORA F4	GHS
**random phenotype**	none	0.00602560	0.00705074	0.00555893
	age, sex	0.00600400	0.00692849	0.00554164
	age, sex, technical	0.00549340	0.00640187	0.00528846
	technical	0.00551280	0.00641387	0.00530522
	technical, PC1	0.00548790	0.00637871	0.00500432
	technical, detected genes	0.00544510	0.00627055	-
	technical, *signal-to-noise ratio*	0.00544820	0.00629034	-
	50 PCs	0.00474210	0.00512433	0.00419344
	age, sex, technical, cell types	0.00542430	-	-
	technical, non-technical	0.00557310	-	-
**BMI**	None	0.00130350	0.00154734	0.00114923
	age, sex	0.00135000	0.00154774	0.00117234
	age, sex, technical	0.00123420	0.00142589	0.00112182
	Technical	0.00119320	0.00142516	0.00109915
	technical, PC1	0.00119210	0.00141686	0.00109480
	technical, detected genes	0.00118540	0.00140998	-
	technical, *signal-to-noise ratio*	0.00119490	0.00141395	-
	50 PCs	0.00125360	0.00126477	0.00105583
	age, sex, technical, cell types	0.00123254	-	-
	technical, non-technical	0.01305295	-	-

50 PCs: the first 50 principal components (PCs) of the principle component analysis (PCA) over the gene expression levels; BMI: body mass index in [kg/m^2^]; cell types: percentage of lymphocytes, neutrophils, monocytes, eosinophils and basophils; detected genes: *number of detected genes* (detection p-value<0.01); Mean SE: mean standard error of phenotypes' beta from all probes of the corresponding association analysis; non-technical: all non-technical parameters having an *Eigen-R^2^* value>0.3% in SHIP-TREND; PC1: the first PC of the PCA; random phenotype: the random phenotype ∼N (0,1); technical: *RNA amplification batch*, *RNA integrity number (RIN)*, *storage time*.

A dash indicates that the parameter was not available in the cohort.

### Effects of SNPs located within oligonucleotides of the probes

SNPs localized within mRNA regions complementary to probes cause base-pairing mismatches, which may result in decreased hybridization efficiency and reduced probe-specific signal intensities. In order to systematically investigate whether these mismatches decrease the binding efficiency of a probe, we analyzed 8898 probes of the HumanHT-12 v3 BeadChip array. Each probe was selected because it covered exactly one exon and could be uniquely mapped to a known transcript listed in the UCSC database [36], [37]. Altogether, 3376 (38%) of these transcripts contained at least one SNP that was included in the 1000 genomes reference panel. When considering the 986 genotyped SHIP-TREND samples, 24% of these regions included a polymorphic SNP, whereas 7% and 4% of these probes contained a SNP with a minor allele frequency (MAF) greater than 0.01 and 0.05, respectively. A subset of 2128 SNPs was used for regression of the probes' expression levels on the number of mismatching alleles per transcript, resulting in 2148 tests due to overlapping probes.

**Figure 3:**
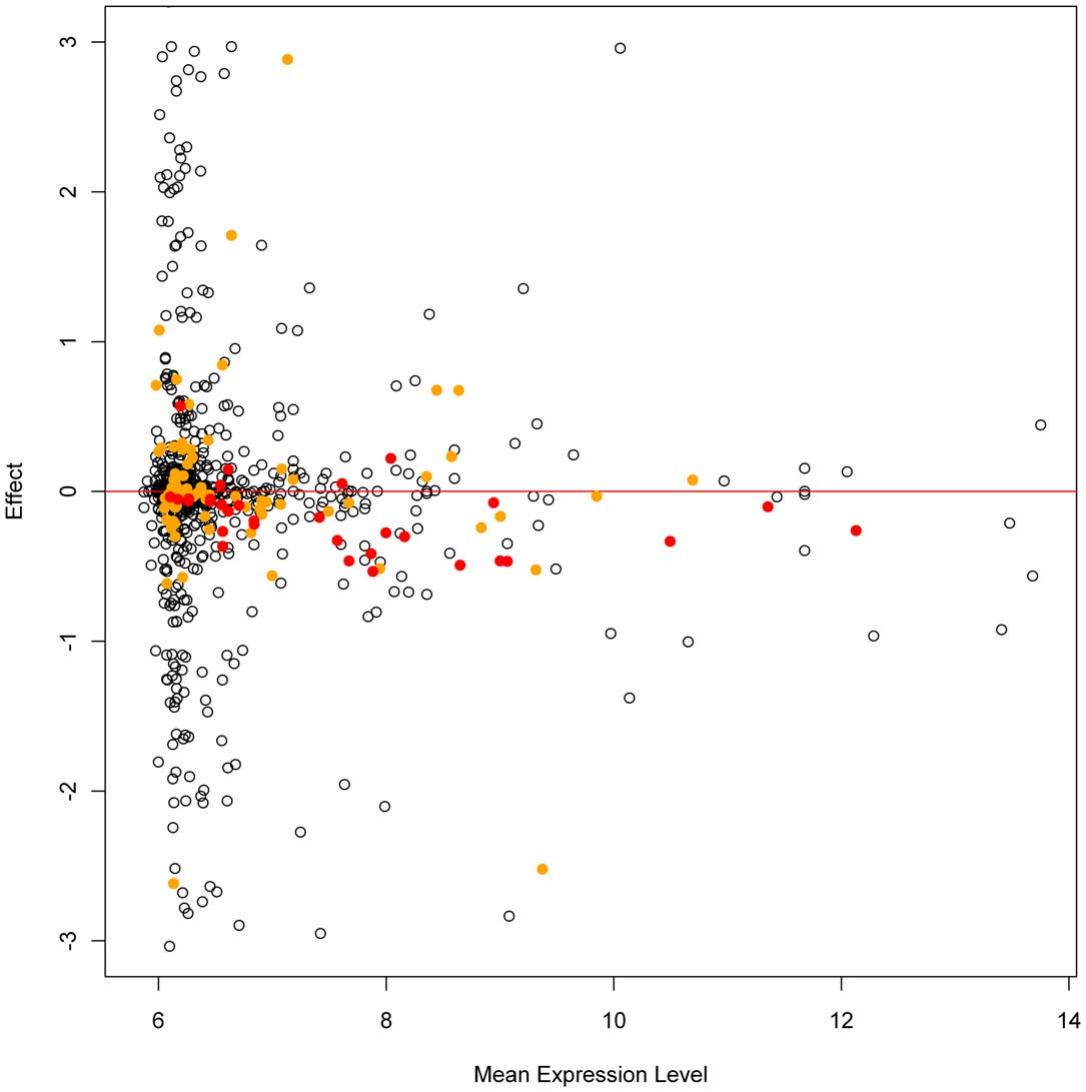
Effects of SNPs within probes on signal intensities. The effects on measured log_2_ transformed (L2T) gene expression levels per mismatch allele of SNPs located within probes (y-axis) are plotted against the mean L2T expression level of the samples for each probe (x-axis). Each spot represents a SNP-probe combination; associations with significant p-values after Bonferroni correction (p<2.3×10^−5^) are colored in red and p-values below 0.05 are colored in orange. To increase legibility the y-axis was limited from −3 to 3 excluding 176 non-significant results out of 1237 successful association results (minimum and maximum effect sizes were −174.1 and 188.7, respectively). Surprisingly, in almost 45% of the associations a positive effect per mismatch allele on expression signal intensity was observed.

The number of transcripts associated with decreased expression signal intensity per mismatch allele of a SNP was significantly higher than the number of transcripts associated with increased signal intensity. This result was consistent in unfiltered analyses and after filtering by nominal or Bonferroni-corrected p-values<0.05 (one-sided binomial test, p-value<0.001 in all three analyses). Nevertheless, 45% of all tested SNPs were associated with increased signal intensity values per mismatch allele (Figure 3). No significant accumulation of association p-values below 0.05 was observed for probes spanning three or more (maximum five) SNPs (χ^2^-test, p-value = 0.73). Furthermore, among 129 SNPs with an association p-value<0.05 many SNPs had at least one SNP in linkage disequilibrium (LD) (n = 86 for R^2^>0.1 and n = 61 for R^2^>0.5) located within the 100 kb region upstream the transcription start site of the respective gene. After the analyses were performed conditional on the SNP having the highest R^2^ with the probe's SNP, p-values increased for all but 12 (91%) transcripts. Finally, out of the 31 statistically significant associations (Bonferroni corrected p-values<2.3×10^−5^), only 12 remained statistically significant after conditioning for the SNP in LD. These results were neither affected by the relative position of the mismatch allele within the probe sequence nor by a low imputation quality of the SNPs (Figure S6, Table S4).

### Annotation of probes using transcript mapping

Since the probe annotation file (HumanHT-12_V3_0_R3_11283641_A, provided by Illumina) was outdated, a new annotation was created based on matching of the probes' sequences to known transcripts listed in the UCSC database (12/06/2009) and by alignment on the human DNA sequence (build HG18 and HG19). The new annotation file for the HumanHT-12 v3 BeadChip array includes 28,961 (59%) probes that perfectly mapped to known transcripts or annotated RefSeq genes and were aligned to build HG19 (Table S5). The chromosomal position as provided in the manufacturer's annotation file (HumanHT-12_V3_0_R3_11283641_A) was unambiguously assigned to coordinates of build HG18 and HG19 for 72% and 28% of uniquely mapped probes, respectively.

## Discussion

Our study showed that the reliability of gene expression analyses is greatly improved by the adjustment for technical factors, which were in particular the *RNA amplification batch*, *RIN* and sample *storage time*. Larger intensity values tend to have higher variance than lower intensity values when repeatedly measured [32]. To be able to perform a linear regression analysis, it is necessary to remove this heteroscedasticity. This can be achieved by applying a logarithm-based transformation on the expression values. A common transformation method used is the L2T, but recently the VST was developed to improve the reduction of the heteroscedasticity, especially in the lower signal intensity range [32]. As pointed out by Schmid *et al.* [38], VST was originally validated on a pre-released version of the HumanRef-8 v1 BeadChip array (Illumina), which differs considerably from the Illumina HumanHT-12 v3 BeadChip array used in the present study and in the work by Schmid *et al.* [38]. Based on the analysis of HaCaT cell expression values, these authors pointed out that VST was outperformed by other methods for the Illumina HumanHT-12 v3 BeadChip array (e.g. L2T) [38]. Although previously reported differences within the lower expression level range [32] could be confirmed in our study by analyzing mRNA signal intensities obtained from human whole blood cells and monocytes using the HumanHT-12 v3 BeadChip array, these differences did not affect the expression association results. Even though both, the VST and L2T, performed well, no remarkable advantage of the VST was observed in our study. Therefore, we decided to perform this and future analyses using the commonly applied L2T instead of VST gene expression data.

**Figure 4:**
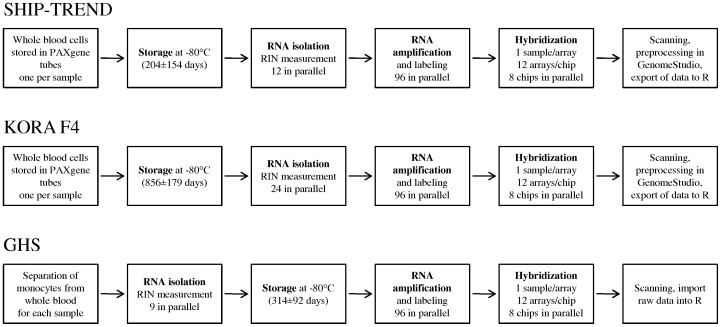
Workflow – from blood sampling to measured mRNA intensities. From left to right: Whole blood was collected and stored in PAXgene tubes until isolation of RNA from whole blood cells in both SHIP-TREND and KORA F4. In GHS, monocytes were separated from whole blood and RNA was isolated from monocytes within 24 hours after blood sampling, subsequently storing the isolated RNA until amplification. The sample *storage time* refers to the duration the whole blood (SHIP-TREND and KORA F4) or isolated RNA (GHS) was stored before further processing, shown as mean ± standard deviation in days. The samples were processed in 96 well plates both after isolation and amplification of the RNA. The corresponding plate layouts were called *RNA isolation batch* and *RNA amplification batch*, respectively. Finally, the RNA was hybridized and the arrays were scanned, quality controlled and analyzed.

PCA revealed that the first PC explained more than 95% of the total variation in gene expression levels. The strong impact of the first PC was consistently found in both cohorts using whole blood cell samples and in the cohort using monocyte samples (Figure S3). We expected to observe consistent results in the two cohorts using whole blood samples because they were obtained from the same tissue type and were processed according to identical protocols in the same laboratory. The additional conformity with the results from the cohort using monocyte samples was surprising, because monocyte samples were handled differently from whole blood samples with respect to several important technical aspects including the *time between blood donation and RNA isolation* as well as the *time between RNA isolation and RNA amplification* (Figure 4). To take these differences into account, we used different parameters to define the sample *storage time* for whole blood cell and monocytes samples. Of note, after adjusting for the technical parameters *RNA amplification batch*, *RIN* and sample *storage time*, the additional adjustment for the first PC lead only to a marginal reduction of the variability of the association results (Table 3), indicating that much of the variation in gene expression signals represented by the first PC may be explained by these three technical factors. Furthermore, the above mentioned technical parameters had the strongest influence on the overall expression signal intensities besides the factors *Illumina chip*, *RNA isolation batch* and the *signal-to-noise ratio*. Almost all samples belonging to the same chip (*Illumina chip*) or 96 well plate after RNA isolation (*RNA isolation batch*) were also processed together on the same 96 well plate after RNA amplification (*RNA amplification batch*). Therefore, adding the parameters *Illumina chip* or *RNA isolation batch* to the regression model did not account for additional variation in this study. Furthermore, one HumanHT-12 v3 BeadChip includes 12 single arrays, each targeting one sample, whereas 96 samples can be processed on one amplification plate. Using the BeadChip information instead of the assigned plate layout after RNA amplification for adjustment would add many more factor levels in the model and might therefore be less powerful. In theory, adjustment for the *signal-to-noise ratio* or the *number of detected genes* (detection p-value<0.01) could further slightly reduce the SE (Table 3). On the other hand, adjusting for these two parameters might falsify the association results for the phenotype of interest like BMI as being correlated with both, the *signal-to-noise ratio* and the *number of detected genes* (Pearson correlation coefficient in SHIP-TREND: *r* = −0.14 and *r* = −0.13, respectively).

Investigating the gene expression levels in 24 whole blood cell samples hybridized to the Affymetrix GeneChip® U133plus 2.0 Array, Xu *et al.* [34] found that the top three PCs explained 28.2%, 17.0%, and 10.2% of the total variance, respectively, which differed strongly from the values obtained from the PCA performed in our study. To investigate whether these differences may be related to the much smaller sample size in the study by Xu *et al.* [34], we selected a random subset of 24 samples (66% female) that belonged to two different BeadChips but were processed on the same 96 well plate for RNA amplification. The PCA results in this small subsample were similar to those obtained from the full dataset (Figure S3) with the top three PCs explaining 97.3%, 0.4%, and 0.3% of the total variance, respectively. However, *Eigen-R^2^* results in the subsample were about an order of magnitude larger than those in the complete dataset and similar to those reported by Xu *et al.* [34]. After adjusting for the effects of technical factors on the gene expression signal intensities and re-running the analyses in the full dataset, the first PC, the top 10 PCs and the top 50 PCs explained 5.9%, 24.8% and 38.5% of the total variation, respectively. Under these conditions, the *Eigen-R^2^* results for the biological parameters remained essentially unchanged, except for serum magnesium concentration (Table S2). These data suggest that the reported *Eigen-R^2^* results reported by Xu *et al.* [34] seemed to be overestimated, probably due to the small sample size. Overall, our data suggest that the large contribution of the first PC may be explained by specific technical factors of the Illumina BeadChip arrays.

Among the biological factors showing the most significant correlation with one of the first five PCs were serum electrolyte concentrations including calcium. Electrolytes as constituents of the internal milieu are involved in several important cellular process and might therefore exert an influence on gene expression in general.

Although much of the variation in expression levels was removed by adjusting for technical parameters (*RNA amplification batch*, *RIN* and the sample *storage time*), the variation was further reduced by correcting for the first 50 PCs (Table 3, Figure S5). This approach was used in recent expression quantitative trait locus (eQTL) analyses [39], [33] and is applicable if the variable of interest (i.e. the SNP) is uncorrelated with any of the PCs used for adjustment. The additional reduction of the variance might be used to successfully reveal additional associations of smaller effect sizes. On the other hand, some PCs may correlate with the phenotype of interest, thereby influencing the association results. In many association settings the PCA approach may therefore not be suitable.

In the present study no consistent effects of SNPs located within the sequence of a probe on hybridization efficiency were observed. Effects that did occur were modest to small and may have been caused by *cis*-effects of SNPs in LD with the respective SNPs in the probe. This notion is supported by the results of an eQTL analysis by conditioning on one SNP in LD located within the upstream region of the transcription start site for each probe: when this SNP was included as an additional covariate in the regression model, the eQTL associations lost significance in most cases. Besides possible *cis*-effects, there might be other effects by which SNPs in strong LD with SNPs within probes can alter transcript signal intensities without affecting binding efficiency. Thus, expression levels of gene products may be modulated by polymorphisms in the 3′-untranslated regions (3′-UTRs) of the gene which represents a preferred binding site for regulatory microRNAs (miRNAs). Polymorphisms interfering with miRNA binding sites have been proposed to affect miRNA-dependent regulation, resulting in modification of the stability or/and translation efficiency of the respective mRNA [40]. Furthermore, it is unlikely that the association results between mRNA levels and non-genetic factors will be markedly confounded by SNPs, regardless whether they are located within probes or not, given the small effect sizes of SNPs found in population-based genome-wide association studies. It is thus more likely for SNPs within probes to cause some extra variability in the expression levels measured by these specific probes than to lead to false positive associations. Based on our data, we recommend keeping probes with SNPs in their sequence for further analyses.

The different chromosomal builds used for probe coordinates as provided in the Illumina HumanHT-12 v3 annotation file might affect the definition of *cis*- or *trans*-eQTL SNPs and must therefore be taken into account when analyzing eQTL effects. Our study shows that the exclusion of probes containing known SNPs would remove a large proportion of probes but does not markedly influence the association results.

In summary, we investigated the influence of data preprocessing and technical effects from sample processing on mRNA signal intensities. We conclude that the adjustment for technical effects can substantially improve the association results, but there is no need to exclude probes containing SNPs. Additionally, we provide a probe annotation file based on transcript mapping and HG19 chromosome build coordinates. In the future, the standardized data preprocessing and analysis platform for measurements of gene expression levels in blood cells as described in this report will be used within the DZHK-MetaXpress consortium to further facilitate meta-analyses of gene expression studies and to explore the expression profile of blood cells associated with several clinical parameters.

## Materials and Methods

### Ethics statement

The study followed the recommendations of the Declaration of Helsinki. The study protocol of SHIP, KORA and GHS was approved by the medical ethics committee of the University of Greifswald, the Bavarian Chamber of Physicians (Bayerische Landesärztekammer) and the federal data safety commissioners (Ethik-Kommission der Landesärztekammer Rheinland-Pfalz), respectively. Written informed consent was obtained from each of the study participants.

### Description of the samples

The Study of Health in Pomerania (SHIP) is a population-based project in West Pomerania, a region in the northeast of Germany. For this project, samples from the SHIP-TREND study were used. Baseline examinations for this study started in 2008. From the total population of West Pomerania comprising approximately 210,000 inhabitants, a stratified random sample of 8016 adults was drawn. Stratification variables were age, sex, and city/county of residence. Stratification variables were age, sex, and city/county of residence. By the end of 2012, approximately 5000 participants will have been examined. Study design and sampling methods were previously described [28].

KORA (Cooperative Heath Research in the Region of Augsburg) exists since 1996 in the region of Augsburg in the southwest of Germany, and builds on the MONICA (Monitoring of trends and determinants in cardiovascular disease) project initiated in 1984 [29]. KORA is a regional research platform for population-based surveys and a cohort of more than 18,000 subjects are actively followed up to date. Four cross-sectional health surveys have been performed in five-year intervals, each containing independent random samples of residents in the city of Augsburg and the two adjacent counties in the age-range between 25 to 74 years at baseline examination. Between 2006 and 2008 6640 samples were collected for KORA F4.

The Gutenberg Health Study (GHS) is designed as a community-based, prospective, observational, single-center cohort study in the Rhine-Main area of Western Germany [41]. The sample was drawn randomly from the governmental local registry offices in the city of Mainz and the district of Mainz-Bingen. The sample was stratified 1∶1 for sex and residence (urban and rural) and in equal strata for decades of age. Individuals between 35 and 74 years of age were enrolled. Exclusion criteria were insufficient knowledge of the German language and physical or psychological inability to participate in the examinations at the study center. Baseline examination of 15,000 study participants was performed between 2007 and 2012.

### Sample selection and preparation

The present analysis is based on a subset of SHIP-TREND participants, and a subset of all KORA F4 participants aged 62 to 81 years [42]. For the first 1001 SHIP-TREND probands that fasted for at least 10 hours prior to blood sampling and had serum fasting glucose levels ≤8 mmol/l and 1002 randomly selected KORA F4 probands, RNA was prepared from whole blood collected and stored in PAXgene tubes (BD, Heidelberg, Germany) using the PAXgene Blood miRNA Kit (Qiagen, Hilden, Germany). Isolation of RNA was performed using a QIAcube according to protocols provided by the manufacturer (Qiagen) in SHIP-TREND and manually in KORA F4. Purity and concentration of RNA were determined using a NanoDrop ND-1000 UV-Vis Spectrophotometer (Thermo Scientific, Hennigsdorf, Germany). To ensure a consistently high RNA quality, all preparations were analyzed using a 2100 Bioanalyzer and RNA 6000 Nano Lab Chips (both from Agilent Technologies, Santa Clara, CA, USA) according to the manufacturer's instructions. Samples exhibiting a *RIN* less than seven were excluded from further analyses. Altogether 17 RNA samples from KORA F4 with automatically called *RIN* values less than seven were included after manual adjustment. Using the Illumina TotalPrep-96 RNA Amp Kit (Ambion, Darmstadt, Germany), 500 ng of RNA were reverse transcribed into cRNA and thereby labelled with biotin-UTP. Hybridization of 3000 ng of labelled cRNA to the Illumina HumanHT-12 v3 Expression BeadChip arrays was followed by washing steps as described in the Illumina protocol. After isolation and quality control processing of all SHIP-TREND and KORA F4 RNA samples was performed at the Helmholtz Zentrum München.

In addition, the present analysis includes a sample of 1374 GHS subjects successively enrolled from April 2007 to April 2008. Blood sampling was carried out under fasting conditions (overnight fast of at least eight hours). Total RNA was isolated from monocytes within 24 h to ensure rapid sample processing. Separation of monocytes from whole blood and preparation of RNA were performed as described previously [30]. The integrity of the total RNA was assessed through analysis on an Agilent Bioanalyzer 2100 (Agilent Technologies, Böblingen, Germany). Samples with a *RIN* less than seven were excluded from further analyses. Gene expression analyses were performed using the Illumina HumanHT-12 v3 BeadChip. RNA samples were processed in 96 well plates. Reverse transcription of 200 ng total RNA and cRNA synthesis with simultaneous biotin labeling were performed using the Illumina TotalPrep-96 RNA Amplification Kit (Ambion, Darmstadt, Germany). Hybridization of 700 ng of each biotinylated cRNA sample with a single array on the BeadChip was performed at 58°C for 16–18 hours. BeadChips were scanned using the Illumina Bead Array Reader.

### Expression data transformation and quality control

In SHIP-TREND and KORA F4 the GenomeStudio V 2010.1 Gene Expression Module was used to impute missing values and for quality control. In detail, samples with less than 6000 significantly detected probes (p<0.01) were excluded (SHIP-TREND: n = 10, KORA F4: n = 4). Subsequently, the probe level data were exported to the R environment for further processing. In GHS, the Illumina raw data files were imported directly into R.

Quantile normalization and L2T was performed in R using the lumi package from the Bioconductor open source software (http://www.bioconductor.org/). Based on expression patterns of probes localised on the X and Y chromosome, we discarded samples which did not match the recorded sex. After quality control, expression data were available for 991 SHIP-TREND, 993 KORA F4 and 1374 GHS samples.

### Principal component analysis

We used a PCA to decompose the variation of the samples' expression profiles into a set of uncorrelated variables of lower dimensions called PCs, with the first PC accounting for the largest part of the total variation of the expression profiles and the subsequent PCs explain less in decreasing order.

### Phenotype definition and covariate adjustment

In total, 66 technical and biological factors were available in SHIP-TREND and selected for initial analyses (Table S2). Of these, all parameters explaining more than 0.3% of the variance of gene expression levels estimated by the *Eigen-R^2^* algorithm were included in subsequent analyses in all three cohorts, if available. Additionally, we included the C-reactive protein (CRP), which was not available in SHIP-TREND, for analyses in KORA F4 and GHS, because it is known to be associated with the overall gene expression levels. The descriptive statistics of these variables are given in Table 1 and Table S1. Regression analyses were performed using gene expression level as dependent variable and the phenotype of interest together with covariates as independent variables in a multiple linear regression model. The mean SEs presented in the analyses of different covariate adjustments were calculated from the corresponding association results of all probes. The analyzed phenotypes were BMI, representing a measure of clinical relevance that is known to influence the overall gene expression profile in blood cells [34], and a pseudo-phenotype artificially generated by selecting random values from a normal distribution being free of any correlation with or confounding by other factors. The random phenotype was used for optimizing variance reduction.

### Annotation of probes by transcript mapping

To validate the binding efficiency of the oligonucleotide probes represented on the Illumina HumanHT-12 v3 BeadChip array, the transcript sequences derived from the 48,803 probe sequences provided in the Illumina annotation file (HumanHT-12_V3_0_R3_11283641_A, version 3.0, 7/1/2010) were mapped against all available mRNA sequences provided in the UCSC genome annotation database (version 12/06/2009, Feb. 2009 assembly of the human genome, HG19) using string matching. Altogether 29,691 probes were successfully mapped to one or more validated mRNAs, which corresponds to previously reported results [43], [44]. Probes that could neither be mapped to a unique mRNA nor to a single annotated RefSeq gene using the UCSC database were flagged accordingly in the annotation file. In total, 28,961 probes (59.3%) were unambiguously associated with a single mRNA or gene. The annotation file was updated using the information obtained by this mapping procedure.

### Genotype calling, imputation and quality control

Genotyping of the SHIP-TREND probands (n = 986) was performed using the Illumina HumanOmni2.5-Quad BeadChip array. DNA from whole blood was prepared using the Gentra Puregene Blood Kit (Qiagen, Hilden, Germany) according to the manufacturer's protocol. Purity and concentration of DNA were determined using a NanoDrop ND-1000 UV-Vis Spectrophotometer (Thermo Scientific). The integrity of all DNA preparations was validated by electrophoresis using 0.8% agarose-1x TBE gels stained with ethidium bromide. Subsequent sample processing and array hybridization were performed as described by the manufacturer (Illumina) at the Helmholtz Zentrum München. Genotypes were called within GenomeStudio with the GenCall algorithm of the Genotyping Module v1.0. Arrays with a call rate below 94%, duplicate samples as identified by estimated identity by descent (IBD) analysis as well as individuals with reported vs. genotyped gender mismatch were excluded. The final sample call rate was 99.51%. Imputation of autosomal genotypes in the SHIP-TREND cohort was performed with the software IMPUTE v2.1.2.3 against the 1000 Genomes Phase I (interim) reference panel released June 2011 (all ancestries panel, build 37). Altogether 667,424 SNPs were excluded before imputation (monomorphic SNPs, Hardy-Weinberg equilibrium p-value≤0.0001, call rate ≤0.9, mapping problem from build 36 to 37) and 88 SNPs were removed after imputation due to duplicate SNP IDs but different positions. The total number of SNPs after imputation and quality control was 37,434,668. The genetic data analysis workflow was created using the Software InforSense. Genetic data were stored using the database Caché (InterSystems, Cambridge, USA).

### SNP-to-transcript matching and association analysis

To assess whether SNPs located within probes could affect the expression signal per mismatch allele due to reduced hybridization efficiency, a suitable subset of probes was selected. Of all probes that could be mapped to a single mRNA transcript as described before and that spanned only one exon, those with missing chromosome or position information of the oligonucleotides were excluded. Probes that mapped to a different chromosome than reported by the manufacturer were also excluded. Of the resulting 8898 oligonucleotides, 72% were annotated using human genome build HG18 coordinates and were mapped to HG19 coordinates using Liftover (http://genome.ucsc.edu/cgi-bin/hgLiftOver). After excluding 163 SNPs for which none of the SNP alleles matched the probe allele at the corresponding position, all 1561 probes located on the DNA forward strand were used for association analysis. Altogether, these probes contained 2128 SNPs with are included in the 1000 genomes reference panel. The analysis of the effect of SNPs on the respective probe's expression level was performed in SHIP-TREND using a linear regression model adjusted for sex, age and the first 50 PCs using the software R [45].

## Supporting Information

Text S1 **Statistical approaches for comparing whole blood cell and monocyte gene expression levels using aggregated data.**
(DOCX)

Figure S1 **Log_2_ transformation (L2T) versus variance-stabilizing transformation (VST): Comparison of mean expression values.** The mean L2T gene expression values (x-axis) are plotted against the mean VST expression values (y-axis) for each probe of the SHIP-TREND (**A**), the KORA F4 (**B**) and the GHS (**C**) cohort, respectively. The L2T data were highly correlated with the VST data for probe intensity values greater than 2^9^. The correlation was recognizably smaller for low probe intensity values.
(TIF)

Figure S2 **Log_2_ transformation (L2T) versus variance-stabilizing transformation (VST): Comparison of association results in KORA F4 and GHS.** The panels show the association results of the random phenotype (A–C) and body mass index (BMI) (D–F) on each gene expression probe adjusted for sex, age, *RNA amplification batch*, *RNA integrity number* (RIN) and the sample *storage time* based on the L2T expression values (x-axis) and the VST expression values (y-axis) in the KORA F4 **(I)** and the GHS **(II)** cohort, respectively. The upper panels (A, D) show the effect sizes (betas), the middle panels (B, E) show the standard errors (SEs) and the lower panels (C, F) show the negative log_10_ association p-values. The corresponding squared Pearson product-moment correlation coefficient between the plotted values is given in the upper right corner of each plot. Each spot represents a probe and is colored according to its mean L2T expression value from all samples. The color code is given in the legend located in the lower right corner of each plot. Despite differing betas and SEs between the two transformation methods, the association p-values obtained with either method were highly correlated.
(TIF)

Figure S3 **Explained Variance of the first 100 principle components (PCs).** (**A**) The cumulated percent of variance (y-axis) explained by the first 100 PCs (x-axis) in SHIP-TREND (blue), KORA F4 (red) and GHS (green) obtained from a principle component analysis (PCA) over the probes using the L2T (crosses) and VST (dots) expression values, respectively. (**B**) The analogous results of the SHIP-TREND cohort using all probes (black) and those excluded by not being significantly expressed above the background level in at least 50% of the samples (grey). Upper left panel: explained variance using L2T; upper right panel: explained variance using VST; lower left panel: explained variance using a subset of 24 samples and L2T expression values; lower right panel: explained variance after computationally removing the influence of technical factors using L2T expression values. In all analyses, except after the adjustment for technical factors, the first PCs explained a high proportion of the total variance.
(TIF)

Figure S4 **Association results of selected factors with the principal components (PCs).** The association results of 26 selected technical and biological factors with each of the first 50 PCs across all three cohorts are shown. Each dot represents an association result, with dot sizes being inversely correlated with the corresponding association p-values. Triangles indicate the PC giving the smallest p-value in each trait and cohort. The PCs are shown on the x-axis. The y-axis represents the traits and cohorts. For each trait, the lower line represents SHIP-TREND (blue), the upper line represents KORA F4 (red) and the middle line represents GHS (green). Grey dots indicate a missing trait in the respective cohort. The PCs were obtained from a principle component analysis (PCA) over the measured gene expression levels. Black dots represent p-values>0.002 (0.05/26 traits). The traits on the y-axis represent the alanine aminotransferase concentrations (ALAT), body mass index (BMI), body weight (WEIGHT), high density lipoprotein concentrations (HDL), hip circumference (HIP), *Illumina chip* (pCHIP), lactate dehydrogenase concentrations (LDH), *month of blood donation* (mDON), *month of RNA isolation* (mISO), *number of detected genes* (DetGene), partial thromboplastin time (PTT), percentage of lymphocytes (Lympho), percentage of neutrophils (Neutro), *RNA amplification batch* (pAMP), *RNA integrity number (RIN)*, *RNA isolation batch* (96 well plate) (pISO), serum calcium concentrations (CA), serum lipase concentrations (LIP), serum magnesium concentration (MG), serum potassium concentrations (K), serum triglyceride concentrations (TG), *signal-to-noise ratio* (StNR), *storage time* (Time), waist circumference (WAIST), white blood cell count (WBC), and vitamin B12 concentrations (B12). While association patterns related to technical factors were similar in all studies (differences in RIN and mDON were related to specific sample processing), the association patterns related to biological factors varied considerably between the studies and even more so between whole blood cells (SHIP-TREND and KORA F4) and monocytes (GHS).
(TIF)

Figure S5 **Standard errors and association p-values using different covariate adjustments.** The figure shows a synopsis of the SEs (lower left of the figure) and the negative log_10_ p-values of the association results (upper right of the figure) for the random phenotype (**A**) and body mass index (BMI) (**B**) based on L2T expression levels in SHIP-TREND. The covariates used in the linear regression models are given in the text panels stretching from the upper left to the lower right. The adjustments used for the x-axis of each scatter plot are specified in the text panel above or below the plot, respectively; the adjustments used for the y-axis of each scatter plot are specified on the left or right, respectively. The spots are colored according to the probes' mean L2T signal intensities, with red representing low and green representing high signal intensities. The principal components (PCs) were obtained from a principle component analysis (PCA) over the expression levels. *PC1* stands for the first PC explaining most of the variation. *Tech* indicates the adjustment for the following technical factors: *RNA amplification batch*, *RNA integrity number* (RIN), and the sample *storage time*. *Cell* represents the white blood cell composition parameters (percentage of lymphocytes, neutrophils, monocytes, eosinophils and basophils, respectively). The strongest reduction of SEs was achieved by adjusting for the first 50 PCs. On the other hand, adjusting for the first 50 PCs resulted in increased p-values for the BMI associations. This effect may have been due to correlations of PCs with BMI. In contrast to its effects on the p-values of the BMI association, adjusting for the first 50 PCs did not substantially affect the p-values of the random phenotype association. Adjusting for the technical factors also substantially decreased the SEs.
(TIF)

Figure S6 **Effects of mismatch alleles within probes on signal intensities.** The negative log_10_ p-values of the association of a SNP located within a probe's sequence on the log_2_ transformed (L2T) gene expression level per mismatch allele are shown on the y-axis. The x-axis represents the position of the SNP in base pairs relative to the beginning of the probe's sequence (**A**) and the SNPs imputation quality (**B**), respectively. Each spot represents a SNP-probe-association. Spots representing associations with significant p-values after Bonferroni correction (p<2.3×10^−5^) appear above the red horizontal line. SNPs with a decreasing effect on the gene expression level are colored in black; SNPs with increasing effect are colored in green. The imputation quality is 0 for poorly and 1 for optimally imputed SNPs. Neither the SNP position within the probe nor the imputation quality significantly affected the association results.
(TIF)

Table S1 **Cohort descriptive of additional parameters.**
(XLSX)

Table S2 ***Eigen-R^2^*** **results for SHIP-TREND of all 66 parameters.**
(XLSX)

Table S3 ***Eigen-R^2^*** **results for SHIP-TREND, KORA F4 and GHS of additional parameters.**
(XLSX)

Table S4 **Association results of SNPs in probe analysis with p-value<0.05.**
(XLSX)

Table S5 **New annotation file for the HumanHT-12 v3 BeadChip array.**
(XLSX)
