# Supplementary material for: Analyzing Illumina Gene Expression Microarray Data from Different Tissues: Methodological Aspects of Data Analysis in the MetaXpress Consortium
Source: PLoS One. 2012 Dec 7;7(12):e50938. doi: 10.1371/journal.pone.0050938 (PMC3517598; doi:10.1371/journal.pone.0050938)
Supplement: Text S1 — Statistical approaches for comparing whole blood cell and monocyte gene expression levels using aggregated data. (DOCX) [file pone.0050938.s001.docx]

# Analyzing Illumina gene expression microarray data from different tissues: Methodological aspects of data analysis in the MetaXpress consortium

## Supplementary Material

**Statistical approaches for comparing whole blood cell and monocyte gene expression levels using aggregated data**

In practice it might be of interest to analyze which genes are differentially expressed between whole blood cell and the monocyte samples depending on the influence of a trait of interest [], whereas often only aggregated data is available for pooled analysis because of data protection regulations. As a consequence, only mean expression intensities as well as effect sizes and SEs of single study association results rather than individual data are available for subsequent analyses.

Hence we tested for mean differences. The null hypothesis using the present data as an example is H_01_: μ_1_ = μ_2_ and H_02_: μ_1_ = μ_3_, where the subscript 1 represents GHS, the monocyte study, and subscripts 2 and 3 represent SHIP-TREND and KORA F4, respectively, which are the whole blood cell samples. If both hypotheses are tested jointly, the test statistic has 2 degrees of freedom (df), and a power loss is anticipated compared to a 1 df test. If the overall test is significant, both single hypotheses can be tested separately without further adjustment for multiple testing. Both whole blood studies had similar sample sizes and their variability was also similar. Their weights in the statistical test should thus be similar and we will use the linear contrast hypothesis H_0_: where μ_i_ represents the tested mean value, and which assumes equal weights for both whole blood studies. We could therefore use weights inversely proportional to the variance of SHIP-TREND and KORA F4 per probe. Assuming similar variances for all three studies, the pooled variance estimator *s*^2^ is given by , where *p_j_* is the number of covariates in the model. The resulting *t*-test statistic is asymptotically standard normally distributed under H_0_.

**References**

[1] Andrew Whitehead and Douglas L Crawford. Variation in tissue-specific gene expression among natural populations. *Genome Biol*, 6(2):R13, 2005.
