# Supplementary figures and images for: Analyzing Illumina Gene Expression Microarray Data from Different Tissues: Methodological Aspects of Data Analysis in the MetaXpress Consortium
Source: PLoS One. 2012 Dec 7;7(12):e50938. doi: 10.1371/journal.pone.0050938 (PMC3517598; doi:10.1371/journal.pone.0050938)

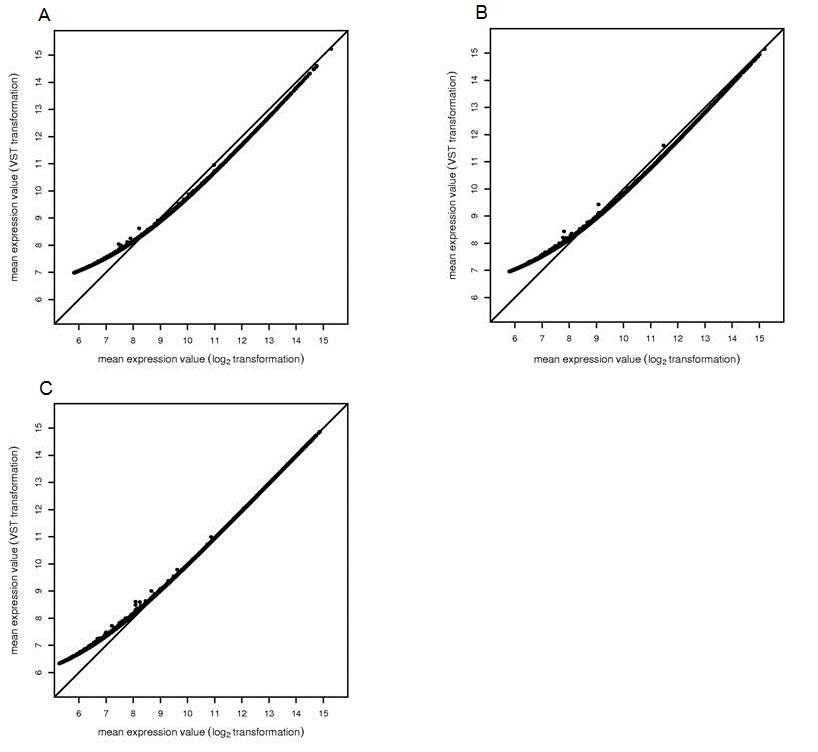

Supplement: Figure S1 — Log2 transformation (L2T) versus variance-stabilizing transformation (VST): Comparison of mean expression values. The mean L2T gene expression values (x-axis) are plotted against the mean VST expression values (y-axis) for each probe of the SHIP-TREND (A), the KORA F4 (B) and the GHS (C) cohort, respectively. The L2T data were highly correlated with the VST data for probe intensity values greater than 29. The correlation was recognizably smaller for low probe intensity values. (TIF) [file pone.0050938.s002.tif]

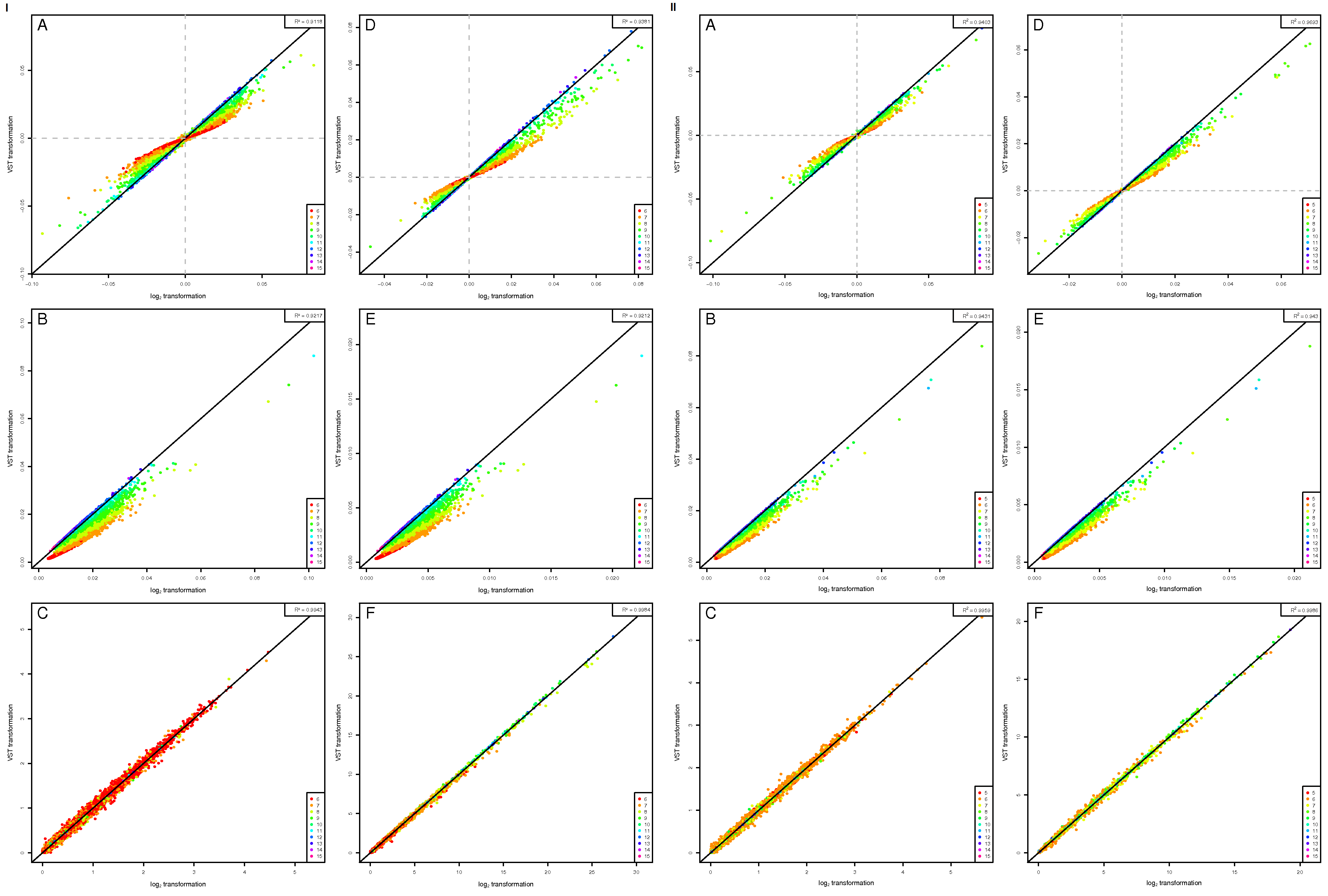

Supplement: Figure S2 — Log2 transformation (L2T) versus variance-stabilizing transformation (VST): Comparison of association results in KORA F4 and GHS. The panels show the association results of the random phenotype (A–C) and body mass index (BMI) (D–F) on each gene expression probe adjusted for sex, age, RNA amplification batch, RNA integrity number (RIN) and the sample storage time based on the L2T expression values (x-axis) and the VST expression values (y-axis) in the KORA F4 (I) and the GHS (II) cohort, respectively. The upper panels (A, D) show the effect sizes (betas), the middle panels (B, E) show the standard errors (SEs) and the lower panels (C, F) show the negative log10 association p-values. The corresponding squared Pearson product-moment correlation coefficient between the plotted values is given in the upper right corner of each plot. Each spot represents a probe and is colored according to its mean L2T expression value from all samples. The color code is given in the legend located in the lower right corner of each plot. Despite differing betas and SEs between the two transformation methods, the association p-values obtained with either method were highly correlated. (TIF) [file pone.0050938.s003.tif]

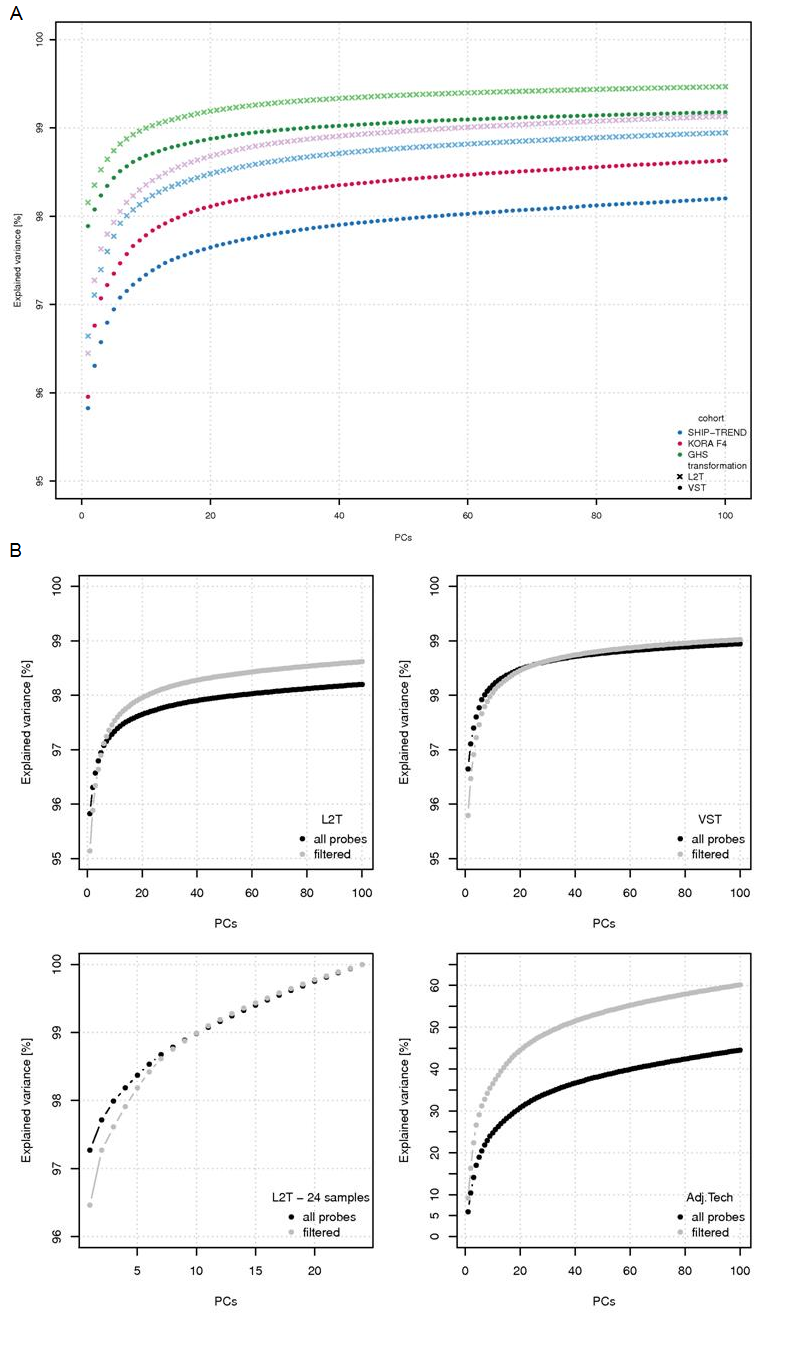

Supplement: Figure S3 — Explained Variance of the first 100 principle components (PCs). (A) The cumulated percent of variance (y-axis) explained by the first 100 PCs (x-axis) in SHIP-TREND (blue), KORA F4 (red) and GHS (green) obtained from a principle component analysis (PCA) over the probes using the L2T (crosses) and VST (dots) expression values, respectively. (B) The analogous results of the SHIP-TREND cohort using all probes (black) and those excluded by not being significantly expressed above the background level in at least 50% of the samples (grey). Upper left panel: explained variance using L2T; upper right panel: explained variance using VST; lower left panel: explained variance using a subset of 24 samples and L2T expression values; lower right panel: explained variance after computationally removing the influence of technical factors using L2T expression values. In all analyses, except after the adjustment for technical factors, the first PCs explained a high proportion of the total variance. (TIF) [file pone.0050938.s004.tif]

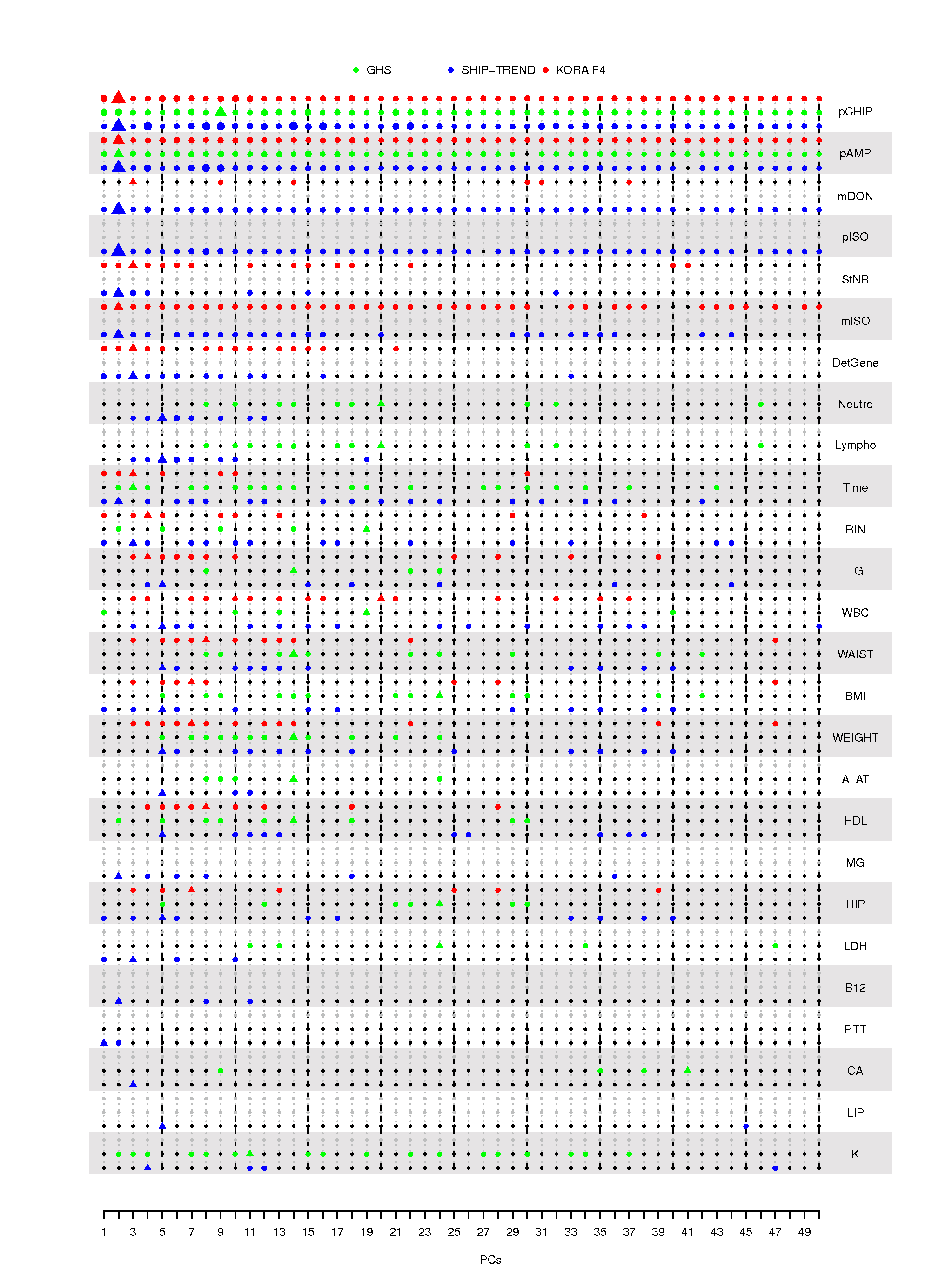

Supplement: Figure S4 — Association results of selected factors with the principal components (PCs). The association results of 26 selected technical and biological factors with each of the first 50 PCs across all three cohorts are shown. Each dot represents an association result, with dot sizes being inversely correlated with the corresponding association p-values. Triangles indicate the PC giving the smallest p-value in each trait and cohort. The PCs are shown on the x-axis. The y-axis represents the traits and cohorts. For each trait, the lower line represents SHIP-TREND (blue), the upper line represents KORA F4 (red) and the middle line represents GHS (green). Grey dots indicate a missing trait in the respective cohort. The PCs were obtained from a principle component analysis (PCA) over the measured gene expression levels. Black dots represent p-values>0.002 (0.05/26 traits). The traits on the y-axis represent the alanine aminotransferase concentrations (ALAT), body mass index (BMI), body weight (WEIGHT), high density lipoprotein concentrations (HDL), hip circumference (HIP), Illumina chip (pCHIP), lactate dehydrogenase concentrations (LDH), month of blood donation (mDON), month of RNA isolation (mISO), number of detected genes (DetGene), partial thromboplastin time (PTT), percentage of lymphocytes (Lympho), percentage of neutrophils (Neutro), RNA amplification batch (pAMP), RNA integrity number (RIN), RNA isolation batch (96 well plate) (pISO), serum calcium concentrations (CA), serum lipase concentrations (LIP), serum magnesium concentration (MG), serum potassium concentrations (K), serum triglyceride concentrations (TG), signal-to-noise ratio (StNR), storage time (Time), waist circumference (WAIST), white blood cell count (WBC), and vitamin B12 concentrations (B12). While association patterns related to technical factors were similar in all studies (differences in RIN and mDON were related to specific sample processing), the association patterns related to biological factors varied [file pone.0050938.s005.tif]

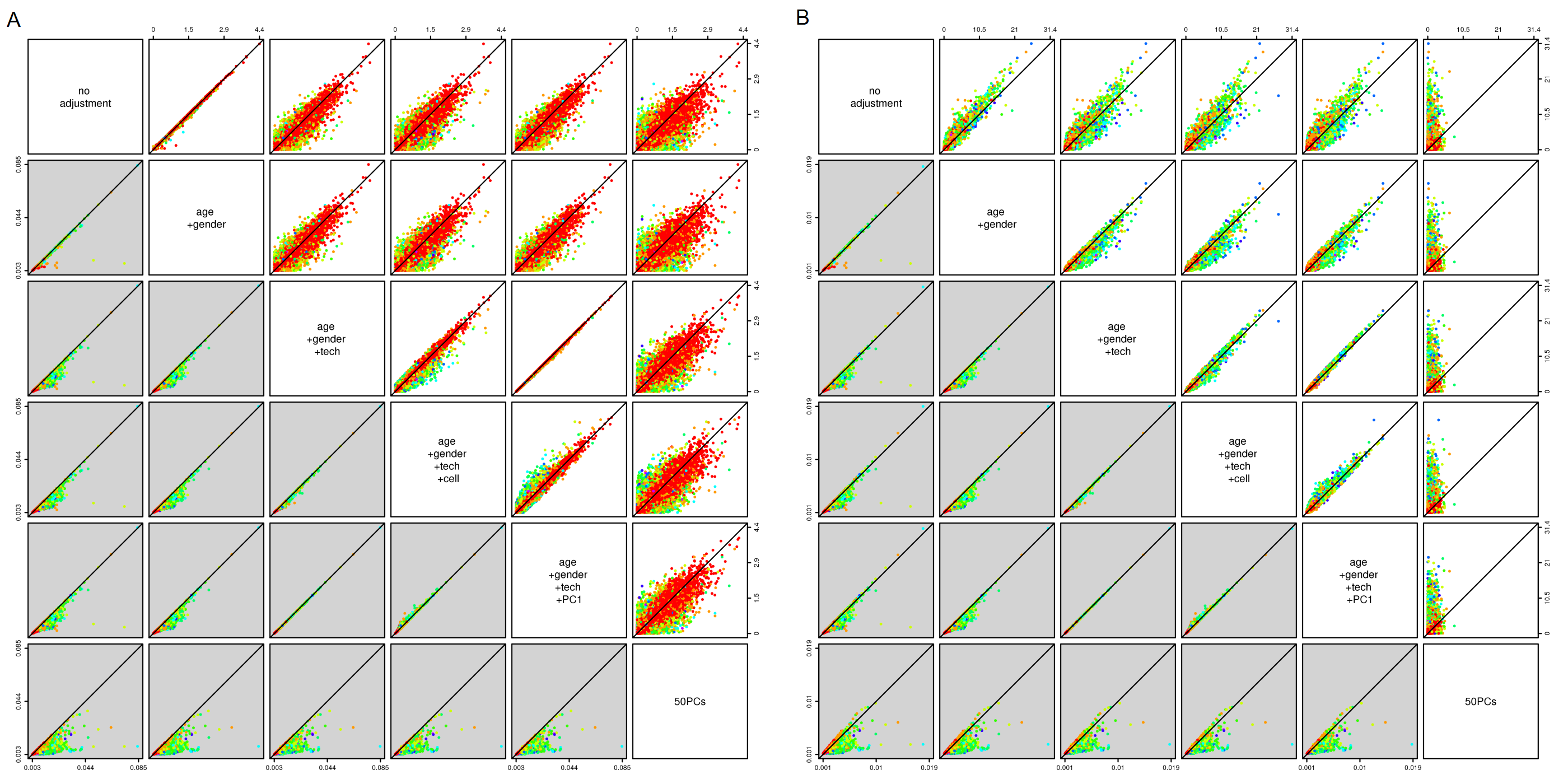

Supplement: Figure S5 — Standard errors and association p-values using different covariate adjustments. The figure shows a synopsis of the SEs (lower left of the figure) and the negative log10 p-values of the association results (upper right of the figure) for the random phenotype (A) and body mass index (BMI) (B) based on L2T expression levels in SHIP-TREND. The covariates used in the linear regression models are given in the text panels stretching from the upper left to the lower right. The adjustments used for the x-axis of each scatter plot are specified in the text panel above or below the plot, respectively; the adjustments used for the y-axis of each scatter plot are specified on the left or right, respectively. The spots are colored according to the probes' mean L2T signal intensities, with red representing low and green representing high signal intensities. The principal components (PCs) were obtained from a principle component analysis (PCA) over the expression levels. PC1 stands for the first PC explaining most of the variation. Tech indicates the adjustment for the following technical factors: RNA amplification batch, RNA integrity number (RIN), and the sample storage time. Cell represents the white blood cell composition parameters (percentage of lymphocytes, neutrophils, monocytes, eosinophils and basophils, respectively). The strongest reduction of SEs was achieved by adjusting for the first 50 PCs. On the other hand, adjusting for the first 50 PCs resulted in increased p-values for the BMI associations. This effect may have been due to correlations of PCs with BMI. In contrast to its effects on the p-values of the BMI association, adjusting for the first 50 PCs did not substantially affect the p-values of the random phenotype association. Adjusting for the technical factors also substantially decreased the SEs. (TIF) [file pone.0050938.s006.tif]

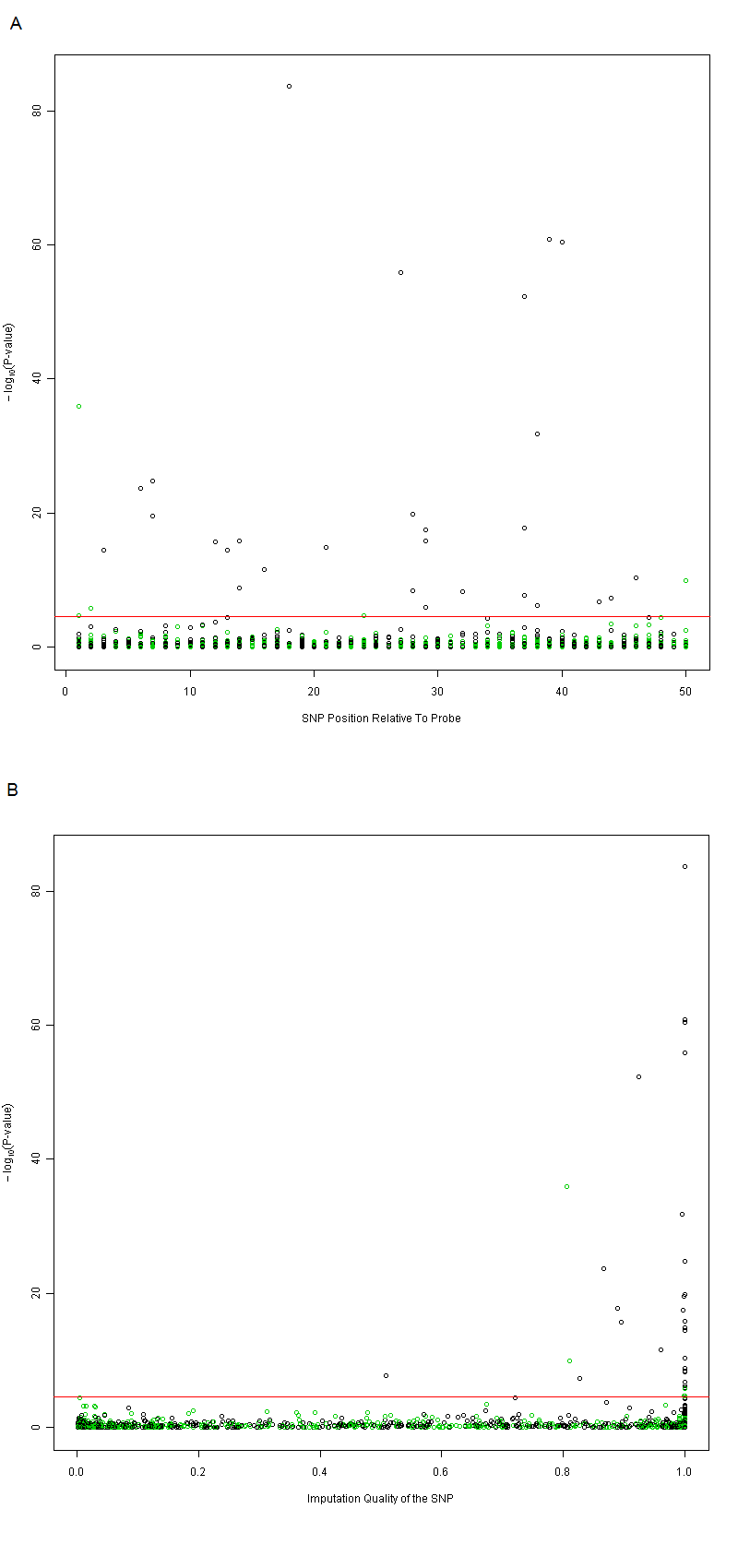

Supplement: Figure S6 — Effects of mismatch alleles within probes on signal intensities. The negative log10 p-values of the association of a SNP located within a probe's sequence on the log2 transformed (L2T) gene expression level per mismatch allele are shown on the y-axis. The x-axis represents the position of the SNP in base pairs relative to the beginning of the probe's sequence (A) and the SNPs imputation quality (B), respectively. Each spot represents a SNP-probe-association. Spots representing associations with significant p-values after Bonferroni correction (p<2.3×10^−5^) appear above the red horizontal line. SNPs with a decreasing effect on the gene expression level are colored in black; SNPs with increasing effect are colored in green. The imputation quality is 0 for poorly and 1 for optimally imputed SNPs. Neither the SNP position within the probe nor the imputation quality significantly affected the association results. (TIF) [file pone.0050938.s007.tif]
